# Ice Nucleation Activity of Perfluorinated Organic Acids

**DOI:** 10.1021/acs.jpclett.1c00604

**Published:** 2021-03-31

**Authors:** Ralph Schwidetzky, Yuling Sun, Janine Fröhlich-Nowoisky, Anna T. Kunert, Mischa Bonn, Konrad Meister

**Affiliations:** † Max Planck Institute for Polymer Research, 55128 Mainz, Germany; ‡ Max Planck Institute for Chemistry, 55128 Mainz, Germany; § University of Alaska Southeast, Juneau, Alaska 99801, United States

## Abstract

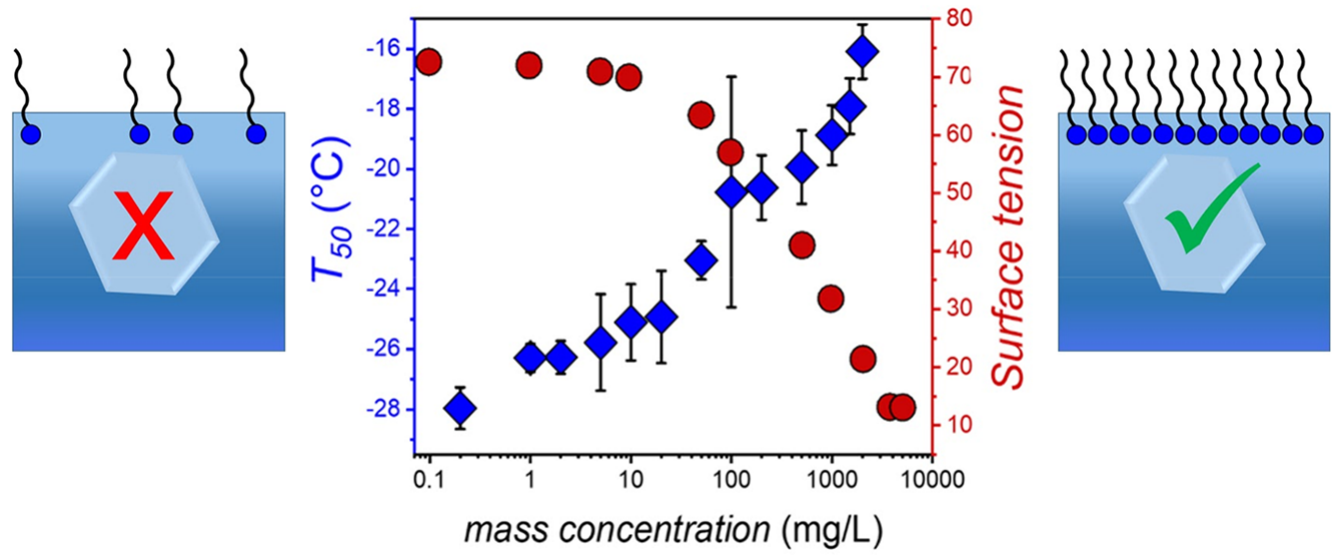

Perfluorinated acids (PFAs) are widely used synthetic chemical compounds, highly resistant to environmental degradation. The widespread PFA contamination in remote regions such as the High Arctic implies currently not understood long-range atmospheric transport pathways. Here, we report that perfluorooctanoic acid (PFOA) initiates heterogeneous ice nucleation at temperatures as high as −16 °C. In contrast, the eight-carbon octanoic acid, perfluorooctanesulfonic acid, and deprotonated PFOA showed poor ice nucleating capabilities. The ice nucleation ability of PFOA correlates with the formation of a PFOA monolayer at the air–water interface, suggesting a mechanism in which the aligned hydroxyl groups of the carboxylic acid moieties provide a lattice matching to ice. The ice nucleation capabilities of fluorinated compounds like PFOA might be relevant for cloud glaciation in the atmosphere and the removal of these persistent pollutants by wet deposition.

Perfluorinated acids (PFAs) such as perfluorooctanoic acid (PFOA) or perfluorooctanesulfonic acid (PFOS) are anthropogenically generated compounds that have emerged as significant global environmental pollutants with persistent, bioaccumulative, and toxic properties.^1,2^ The adverse environmental effects of PFAs have led to their addition to annexe A of the Stockholm Convention for persistent organic pollutants, and PFOS and related chemicals were voluntarily removed from the market.^3^ Despite the efforts to stop the environmental release, products containing PFAs remain in use and continue to contribute to environmental contamination. Of the perfluorinated acids, PFOA is the most ubiquitous pollutant due to its extensive usage in the fluoropolymer industry and high total emissions.^4,5^ PFOA has been observed in different air and water sources (rain, snow, sea) and was detected in regions as remote as the High Arctic.^6,7^ Since there are no primary sources of PFOA in remote locations that could contribute to contamination, questions arise regarding the sources and transport pathways of this concerning pollutant.^7,8^ The currently suggested long-range transport pathways of PFOAs are hydrospheric and atmospheric, with the latter being more relevant for remote locations and the Arctic.^9,10^ This can be witnessed by high PFA and PFOA concentrations in the Arctic atmosphere and ongoing detection of PFOA and PFAs in Arctic snow samples.^7,11^

Within the atmosphere, perfluorinated compounds can undergo atmospheric oxidation and react with Criegee intermediates,^12^ but they could also interact with clouds,^13^ which are important for weather effects due to cloud glaciation and precipitation. Pure water droplets do not freeze homogeneously until ∼ −38 °C owing to the energy barrier associated with creating the initial crystallization nucleus.^14^ In cloud droplets, water typically freezes in a heterogeneous process, facilitated by the presence of particles that serve as ice nucleators (IN). Common abiotic IN include clay, dust, minerals, or carbonaceous materials.^15^ Biogenic IN consist of biomolecules derived from bacteria, fungi, insects, or pollen.^16^ Among the abiotic ice-nucleating surfaces, monolayers of long-chain alcohols have been shown to be particularly effective, while fatty acids with similar chain lengths are significantly less so.^17^ Here, we report that PFOA is an efficient IN, much more so than the structurally similar PFOS and octanoic acid (OA). These compounds consist of a hydrophobic tail and a hydrophilic headgroup, and are known to accumulate and form monolayers at the air–water interface (Figure 1).^18^ The ice nucleation activities of the (fluoro)surfactants are investigated using the high-throughput Twin-plate Ice Nucleation Assay (TINA).^19^ TINA enables the simultaneous measurement of a complete dilution series with each series composed of hundreds of droplets of a few microliters with high statistics, enabling the analysis and characterization of the efficiency of particles with high accuracy.^20−22^

**Figure 1:**
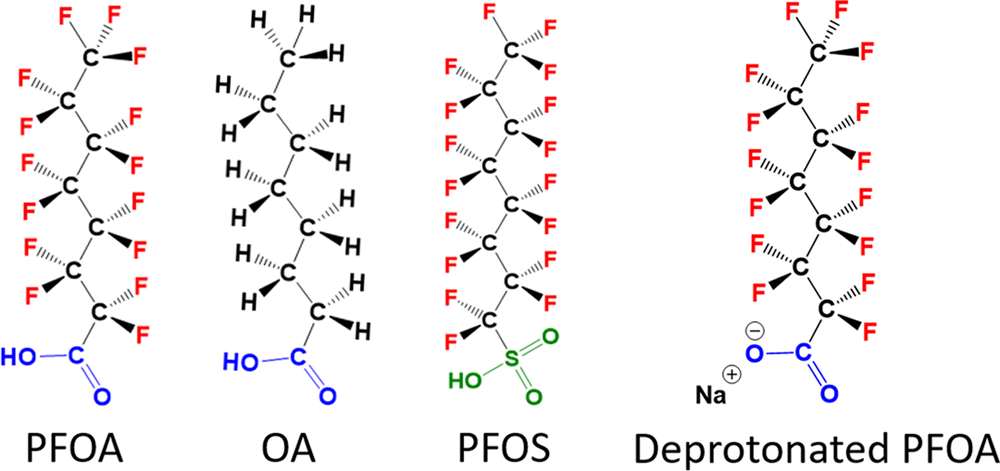
Chemical structures of the investigated perfluorooctanoic acid (PFOA), octanoic acid (OA), perfluorooctanesulfonic acid (PFOS), and deprotonated PFOA.

Figure 2A shows the results of statistical freezing curves of aqueous PFOA solutions with concentrations between 0 and 2000 mg/L, while Figure 2B shows the *T*_50_ values of PFOA solutions as a function of concentration. The *T*_50_ values are defined as the temperatures at which 50% of the droplets are frozen. PFOA shows considerable ice nucleating activity, in a manner highly dependent on the solution concentration.

**Figure 2:**
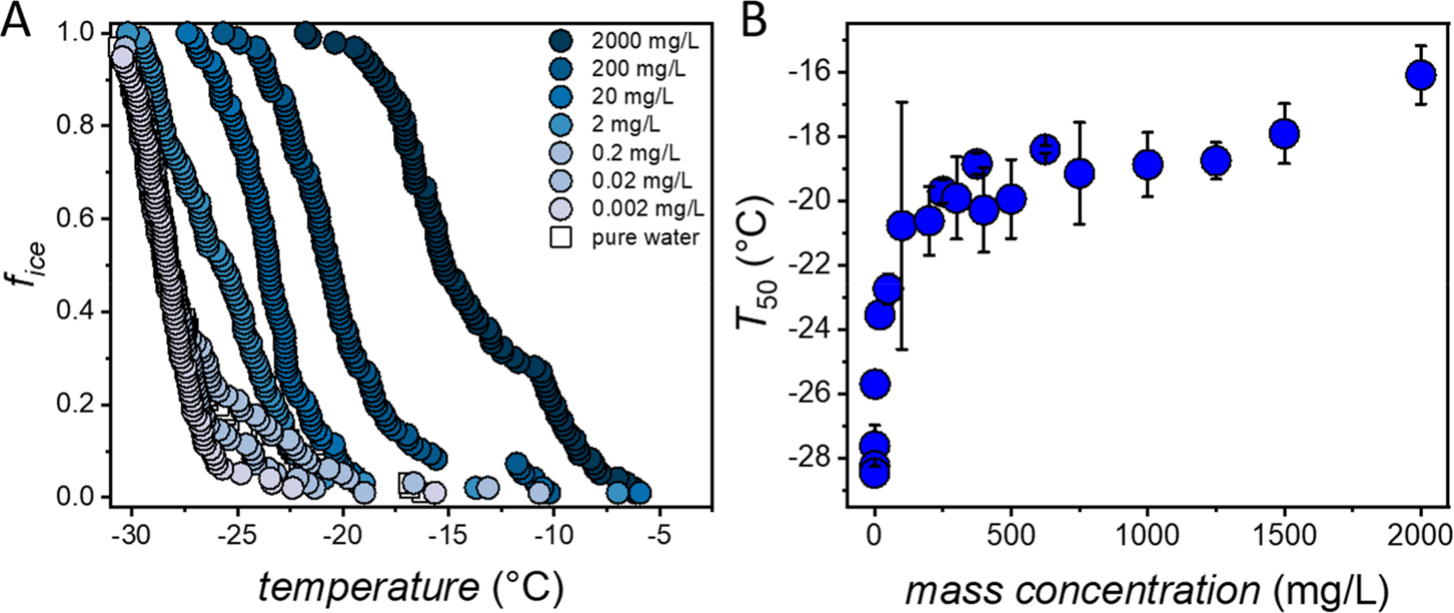
Ice nucleation activity of PFOA. (A) Freezing curves of aqueous PFOA solutions from concentrations ranging from 0 to 2000 mg/L. Shown are the fraction of frozen 3 μL droplets vs temperature. The point at which 50% of the droplets are frozen (*f*_ice_ = 0.5) represents the *T*_50_ value. (B) *T*_50_ values of aqueous PFOA solutions as a function of concentration. Error bars represent the standard deviation of 3–8 independent measurements.

At PFOA concentrations up to 0.02 mg/L, the ice nucleation activity is negligible, with freezing occurring at *T*_50_ = ∼ −28 °C, comparable to pure water in our experimental setup. Increasing the concentration above 0.02 mg/L results in freezing temperatures that are higher than that of pure water. We find that for 200 mg/L PFOA solutions *T*_50_ = ∼ −21 °C, and for 2000 mg/L solutions, *T*_50_ increases up to ∼ −16 °C. While the maximal determined *T*_50_ value is ∼ −16 °C, it is also worth mentioning that we occasionally observed high initial freezing temperatures of up to −5 °C even at low concentrations (Figure 2A).

Interestingly, the *T*_50_ values of the droplet freezing statistics do not simply increase linearly with higher concentration. Instead, the data for PFOA shows resemblance with a Langmuir adsorption model with an initial rapid increase in *T*_50_ up to ∼200 mg/L and a subsequent slower increase and leveling off of the ice nucleation activity until 2000 mg/L.

Next, we determined the ice nucleation activity of OA, PFOS, and deprotonated PFOA to unravel which properties of PFOA give rise to its ice nucleation efficiency. The activities of OA, deprotonated PFOA, and PFOS were determined over different concentration ranges owing to their respective solubilities in water.

Figure 3 shows the *T*_50_ values of OA, deprotonated PFOA, and PFOS solutions plotted as a function of concentration in aqueous solution. We find that, similar to PFOA, the *T*_50_ plots of all three compounds resemble Langmuir adsorption models. However, in contrast to PFOA, the maximal ice nucleation activities were significantly lower for all three (fluoro)surfactants. For OA, we found that the maximal ice nucleation activity is at ∼ −24 °C, which is only slightly higher than the freezing temperature of pure water in our setup. Apparently, the perhydrogenated fatty acid is a significantly poorer ice nucleator than perfluorinated PFOA (Figure S1). For PFOS, the maximal ice nucleation activity was ∼ −20.5 °C, but at 20 times higher concentration than PFOA. Deprotonation of the carboxylic acid headgroup of PFOA eliminates most ice nucleation activity with a maximum of ∼ −26.5 °C. It seems that both changing the hydrophilic headgroup of PFOA or the hydrophobicity of the tail suppresses the ice nucleation activities of the respective (fluoro)surfactants. We performed dynamic light scattering and calorimetric measurements to examine whether different water activities or solution aggregates may be the origin of the observed differences in the ice nucleation capabilities. Neither the melting points of the compounds nor their aggregate sizes were found to differ notably (Table S1, Figure S2), eliminating explanations involving different water activities or aggregate sizes in solution as the origins for the observed differences in ice nucleation activity.

**Figure 3:**
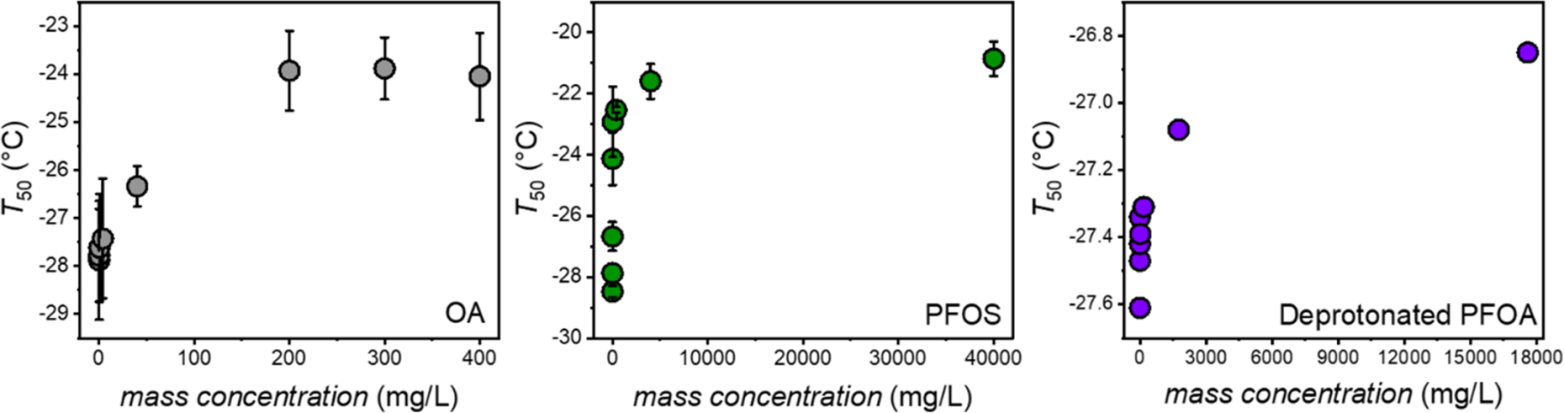
Ice nucleation activity, quantified through *T*_50_ values, of OA (gray circles), PFOS (green circles), and deprotonated PFOA (purple circles) solutions as a function of concentration. Error bars represent the standard deviation of 3–5 independent measurements.

PFOA and other fluorosurfactants are known to accumulate and form monolayers at the air–water interface,^18^ with a maximum surface excess of ∼2 mg/m^2^ for aqueous concentrations exceeding 100 mg/L. In the TINA droplet freezing experiments, the surface pressure cannot be controlled and is a function of the amount of PFOA at the surface and the temperature. Interestingly, we find that the observed ice nucleation activities of PFOA and the other surfactants directly correlate with their surface tensions, implying that their ice nucleation activities are linked to the buildup of the (fluoro)surfactant monolayers (Figure 4B, Figure S3). We exclude the possibility that multilayered structures or micelles form or coexist underneath the PFOA monolayer, since we observe no changes in aggregate size in DLS measurements (Figure S4). The critical micelle concentration for PFOA also falls below the solubility limit, and X-ray reflectivity measurements showed that the thickness of a perfluorinated carboxylic acid layer corresponds to a monolayer state of the film.^23^

**Figure 4:**
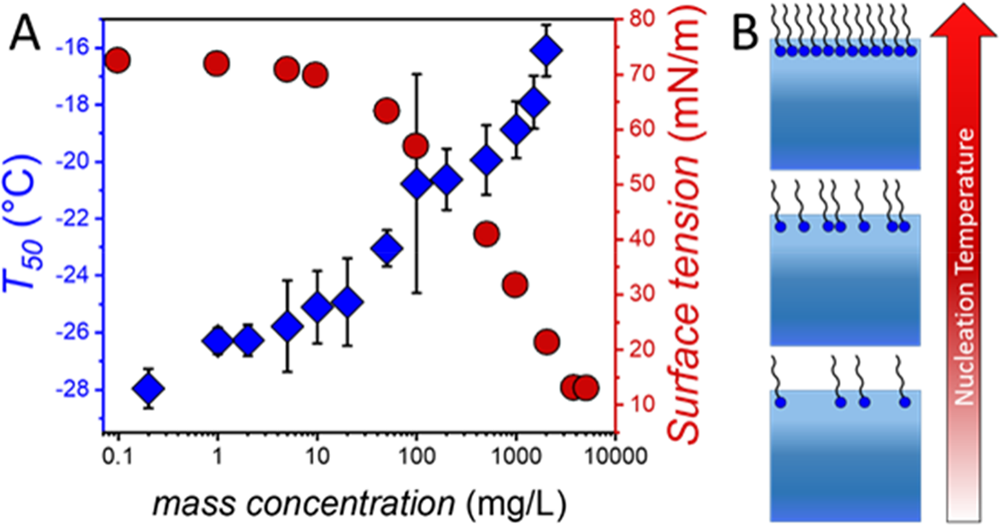
Surface tension and ice nucleation of PFOA. (A) The ice nucleation activity of PFOA is concentration dependent and follows the trend of the surface tension. Surface tension values were derived from Lyu et al.^26^ (B) Schematic representation of the buildup of a PFOA monolayer, which correlates with the increase of the ice nucleation activity (nucleation temperature) of PFOA.

Previously, monolayers of *n*-alkyl alcohols have been shown to be particularly effective in nucleating ice and that their freezing temperatures increased with the length of the hydrocarbon tail.^17^ These monolayers expose hydroxyl groups to water in a manner that resembles the basal plane of ice. Hence, it was suggested that the structural lattice matching with ice governs their ice-nucleating efficiency. Interestingly, fatty acid monolayers, which also expose hydroxyl groups to water, are very poor ice nucleators, with solid fatty acid crystals showing more promise.^24^ Molecular simulations have previously suggested that the discrepancy between the alcohol and fatty acid layers was due to differences in the monolayer’s compactness and the resulting structural match to ice, which are key for determining the ice nucleation ability of organic surfaces that expose hydroxyl groups to ice.^25^

Upon fluorination, hydrophobic chains will undergo structural and conformational changes that directly affect the packing of the monolayer. Structurally, perfluorinated chains display a larger footprint (∼0.28 nm^2^) than hydrogenated chains (∼0.19 nm^2^) and thus lower interfacial densities and molar volumes than hydrogenated chains with the same number of carbon atoms.^27^ There are also conformational differences. For perfluorinated chains, the dihedral angle at minimum energy is not exactly 180°, as it is for hydrogenated ones. Consequently, perfluorinated chains adopt a characteristic helical conformation, while hydrogenated chains tend to be in an all-*trans* planar zigzag form.^28−30^ Moreover, the energy barrier for internal rotation of perfluorinated chains is appreciably higher than for hydrogenated chains, which induce a rigid character, in contrast with the flexible character of hydrogenated chains.^29^

Altogether, the Langmuir monolayers of fluorinated molecules will have a higher crystallinity than their hydrocarbon counterparts. In fact, grazing incidence X-ray diffraction studies of monolayers of perfluorinated carboxylic acids have revealed tight hexagonal packing of molecules with their long axes nearly perpendicular to the water surface and the coexistence of crystalline and dilute disordered phases.^23,31^

We conclude that upon fluorination, the morphology and packing within the monolayer allow for a better alignment of the carboxylic acid groups with less structural fluctuations, thereby providing a better ice template and enabling enhanced nucleation properties. This hypothesis is supported by additional measurements of perfluorodecanoic acid, which also shows good ice nucleation abilities (Figure S5). Our conclusion is also in line with previous suggestions based on MD simulations and experimental findings that solid fatty acid particles are better INs than fatty acid monolayers.^24,25^ Irrespective of the precise molecular mechanism, the finding that PFAs have high ice nucleation activity may have direct implications for the transport and environmental fate of these persistent organic pollutants, as they could get distributed to remote environments by actively being involved in cloud glaciation.

While the local concentration at the anthropogenic point of origin may be high, once it becomes distributed in the environment, average concentrations found in the atmosphere are significantly lower than the ones reported here (∼15 pg/m^3^),^32^ and the deprotonated PFOA form is likely prevalent, which has low ice nucleation activity.^33^ Our results suggest that increasing the crystallinity and order of monolayers through perfluorination will also affect the ice nucleation abilities of other perfluorinated compounds such as long-chain alcohols, potentially rendering them from good to exceptional ice nucleators with direct atmospheric implications.^13,17^ The possible ice nucleation synergy between hydrogenated and perfluorinated long-chain alcohols and the interplay of PFAs with other ice-nucleating particles found in the atmosphere are yet to be investigated.

## Supporting Information Available

Experimental details and supporting Figures S1–S5 (PDF)
