# Supplementary material for: Ice Nucleation Activity of Perfluorinated Organic Acids
Source: J Phys Chem Lett. 2021 Mar 31;12(13):3431–5. doi: 10.1021/acs.jpclett.1c00604 (PMC8040019; doi:10.1021/acs.jpclett.1c00604)
Supplement: Supplementary file 1 — jz1c00604_si_001.pdf [file jz1c00604_si_001.pdf]

Supporting Information:

Ice Nucleation Activity of Perfluorinated Organic Acids

Ralph Schwidetzky[#,†], Yuling Sun[†], Janine Fröhlich-Nowoisky[§], Anna T. Kunert[§], Mischa Bonn[†] and Konrad Meister[†,⊥,]*

[†]Max Planck Institute for Polymer Research, 55128 Mainz, Germany

[§]Max Planck Institute for Chemistry, 55128 Mainz, Germany

[⊥]University of Alaska Southeast, 99801 Juneau, AK, United States

Corresponding Authors

K. Meister − Max Planck Institute for Polymer Research, 55128 Mainz, Germany; University of Alaska Southeast, Juneau, Alaska 99801, United States; orcid.org/0000-0002-6853-6325; Email: meisterk@mpip-mainz.mpg.de

Materials and Methods

Samples: PFOA, PFDeA OA, and PFOS were obtained from Sigma Aldrich. TINA experiments were performed in ultrapure water, which was prepared as described elsewhere[1]. Deprotonated PFOA was obtained by dissolving PFOA in 0.1 M sodium hydroxide (Roth).

TINA Experiments. Ice nucleation experiments were performed using a high-throughput droplet freezing assay. The details of the instrument have been described recently[1]. In a typical experiment, 96 droplets (3 µL) of the investigated solutions were placed on two 384-well-plates by a liquid handling station (epMotion ep5073, Eppendorf, Hamburg, Germany) and tested with a continuous cooling-rate of 1 °C/min from 0 °C to −30 °C. The droplet-freezing was determined by two infrared cameras (Seek Therman Compact XR, Seek Thermal Inc., Santa Barbara, CA, USA). The uncertainty in the temperature of the setup was ±0.2 °C. Samples were measured several times with independent samples (PFOA 8 times, OA 3 times, PFOS 2 times, deprotonated PFOA 2 times).

DSC Measurements. Differential Scanning Calorimetry (DSC 822 with sample robot by Mettler Toledo) was used to determine the melting points. All samples were measured in a range from −60 to 20 °C (heating rate: 1 K/min, cooling rate: −10 K/min). Measurements consisted of two cooling/heating cycles and the corresponding melting points were averaged.

DLS Measurements. Dynamic light scattering (DLS, Submicron Particle Sizer Nicomp 380 with a fixed scattering angle of 90°, laser wavelength $\lambda = 632.8$ nm) was used to determine the sizes of any solution aggregates of the (fluoro)surfactants. DLS measurements were typically performed at concentrations of 0.2 mg/mL.

Surface Tension Measurements. Surface tension was measured using Du Noüy rings (DCAT, DataPhysics Instruments GmbH) and SCAT 32 software. Each concentration was measured twice, and all measurements were performed at 22 °C.

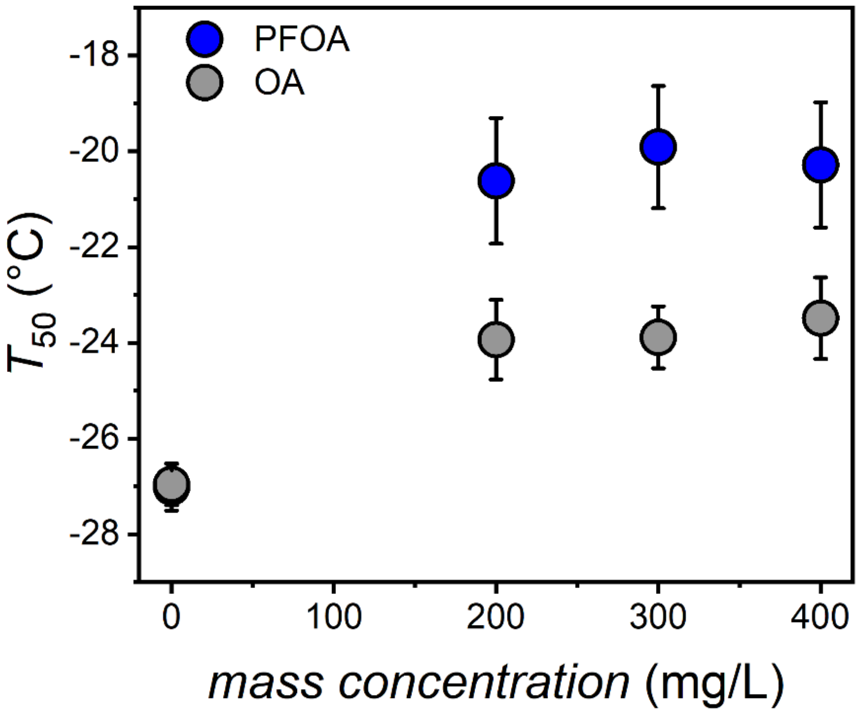

Figure S1*:* Comparative representation of the ice nucleation activity of PFOA (blue) and OA (grey). The values represent the average of three independent experiments.

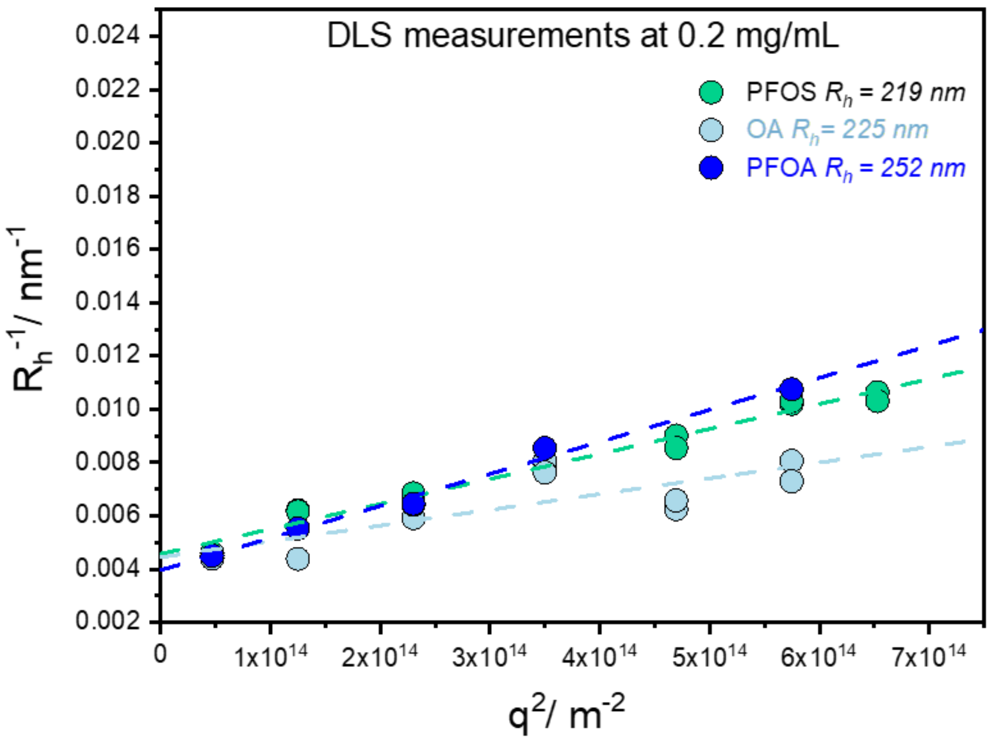

Figure S2*:* Particle sizes of PFOA, OA and PFOS in aqueous solution at concentrations of 0.2 mg/mL as determined by DLS measurements.

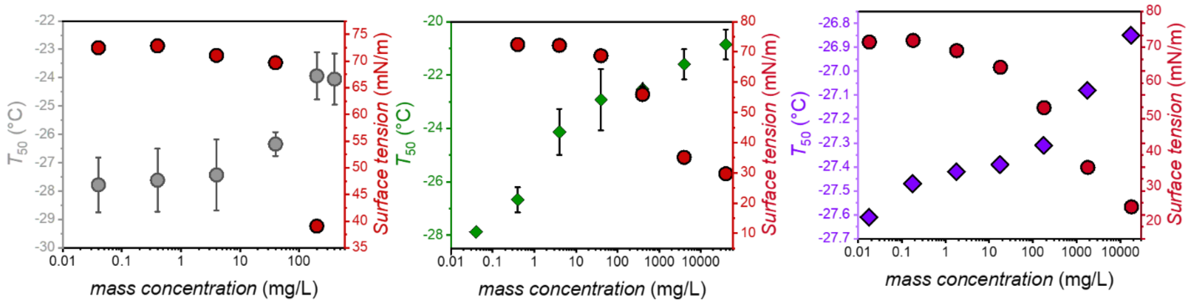

Figure S3*:* Surface dependence of OA (grey), PFOS (green) and deprotonated PFOA (purple). The ice nucleation activity of the three compounds is concentration-dependent and follows the trend of the surface tension (red) similar to PFOA.

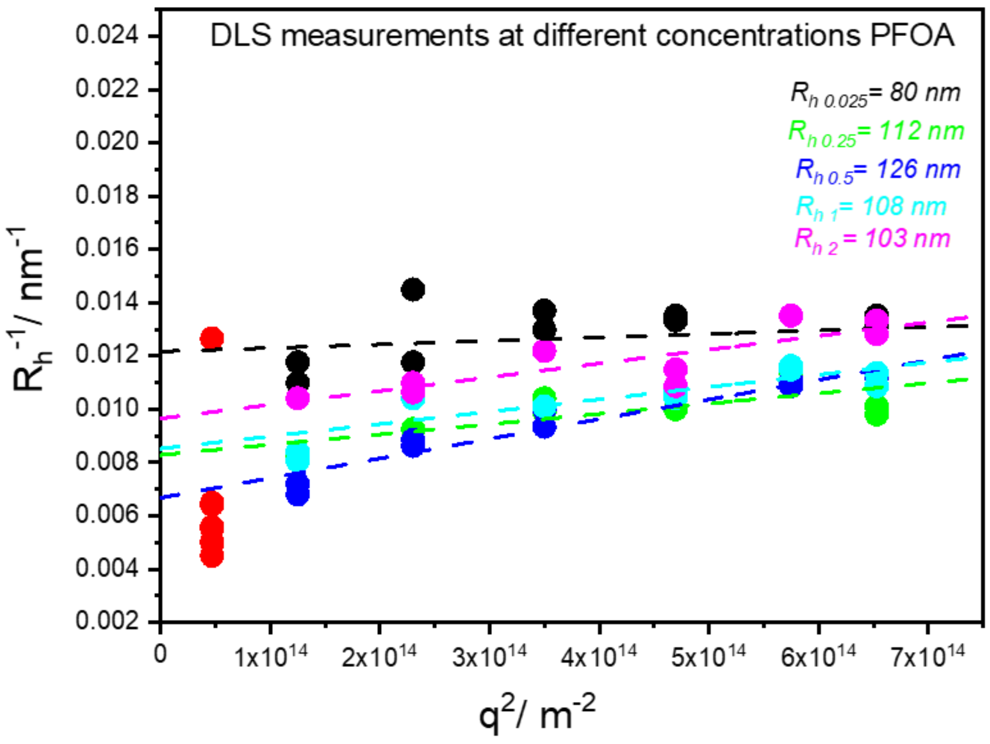

Figure S4: Particle sizes of PFOA in aqueous solution at concentrations spanning from 0.025 mg/mL to 2 mg/mL as determined by DLS measurements.

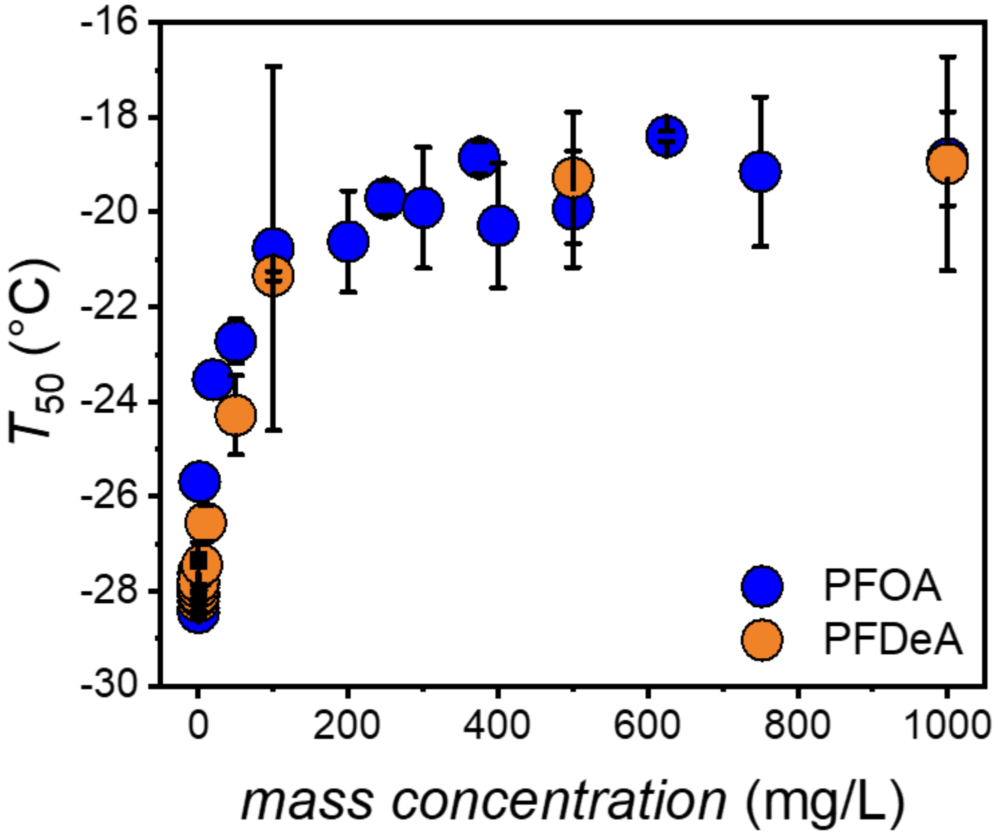

Figure S5*:* Comparative representation of the ice nucleation activity of PFOA (blue) and perfluorodecanoic acid (PFDeA, orange).

Table S1: Melting Points of PFOA, OA and PFOS at concentrations of 0.2 mg/mL in water as determined by DSC measurements.

Sample	Melting Point (°C)
**PFOA**	0.06°C
**OA**	0.09°C
**PFOS**	0.01°C

References

(1.) Kunert, A. T.; Lamneck, M.; Helleis, F.; Pöschl, U.; Pöhlker, M. L.; Fröhlich-Nowoisky, J. Twin-Plate Ice Nucleation Assay (TINA) with Infrared Detection for High-Throughput Droplet Freezing Experiments with Biological Ice Nuclei in Laboratory and Field Samples. *Atmos. Meas. Tech.* **2018,** *11* (11), 6327-6337.
